# Real-Time Acetone Gas Monitoring Using Calixarene-Functionalized Guided-Mode Resonance-Based Sensors

**DOI:** 10.1021/acsphotonics.6c00114

**Published:** 2026-02-24

**Authors:** Adam Rigler, William P. Unsworth, Thomas F. Krauss

**Affiliations:** † School of Physics, Engineering, and Technology, 8748 University of York, York YO10 5DD, U.K.; ‡ Department of Chemistry, University of York, York YO10 5DD, U.K.; § York Biomedical Research Institute, University of York, York YO10 5DD, U.K.

**Keywords:** guided-mode resonance, gas sensor, calixarene, biosensor, acetone, photonic, resonant

## Abstract

Portable sensors to detect volatile organic compounds (VOCs) are needed for many societal and industrial applications, such as diagnosing patient health through breath analysis or monitoring industrial environments for worker safety. Acetone, a widely used industrial solvent, is an important example that requires sensitive detection to prevent hazardous exposure over workplace safety limits. Established sensor technologies suffer from drawbacks such as high operating temperatures and power consumption, long-term drift, or high complexity. Here, we present an optical gas sensor based on a guided-mode resonance (GMR) that affords handheld operation and a simple readout. The GMR sensor features a medium quality (Q-factor) and is functionalized with a calixarene layer that exhibits a refractive index change upon exposure to acetone. The sensor achieves a limit of detection (LOD) of 80 ppm for acetone vapor at room temperature, with a response following an extended Langmuir isotherm, making it highly suitable for monitoring workplace safety, where a prolonged exposure to levels >170 ppm is considered dangerous. We also show that the sensor response is repeatable within a 4.5% standard deviation across measurements, highlighting that the technology offers a low-cost, high-performance solution for monitoring workplace acetone levels.

## Graphical abstract

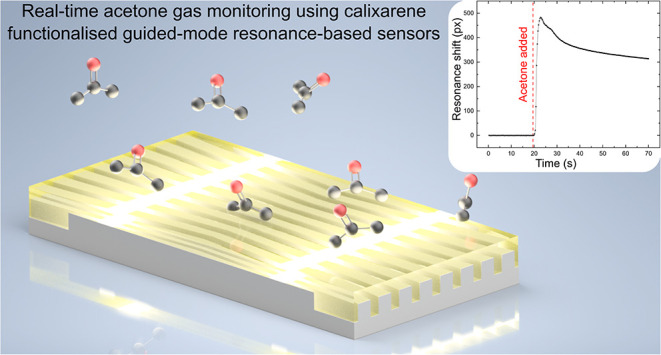

## Introduction

The detection of volatile organic compounds (VOCs), such as ketones, esters, and aldehydes, is important for many applications, including food production, healthcare, and environmental monitoring, as their inhalation can cause significant harm to our health. This issue is particularly relevant to industrial environments, where VOC concentrations are often increased, placing workers and the public at risk. For example, acetone is a VOC that plays a crucial role in many industrial processes, such as the manufacturing of plastics, and is a commonly used precursor for many organic synthesis processes. Due to its low boiling point, acetone readily evaporates at room temperature, and prolonged exposures above 173 ppm can cause damage to respiratory airways and the central nervous system.^1^ Therefore, an accurate monitoring method that can be widely deployed is important, and research is ongoing on novel detection methods.

VOCs are challenging to measure due to their diverse chemical properties, varying concentrations, and mixed sample space, and their volatile nature implies low binding affinities. Unlike in the liquid phase, where antibodies and other recognition molecules are readily available to convey specificity, it is more difficult to achieve specificity with gas-phase measurements. An ideal gas sensor will be highly sensitive, specific, and stable over long periods. In this context, current acetone-sensing technologies, while widely adopted across industrial settings, suffer from a variety of limitations.

### Current Sensing Technologies

Metal-oxide-semiconductor (MOS) sensors are the most commonly used modality, operating via a chemiresistive interaction.^2^ Although inexpensive to produce and offering high sensitivity (with limits of detection in the high ppb region), these sensors have poor selectivity, requiring high operating temperatures and therefore high power consumption, and suffer from long-term drift.^3,4^

Similarly, electrochemical sensors operate by measuring changes in either current, potential, or conductivity across two electrodes separated by a chemical cell.^5^ This method of operation offers superior selectivity and lower power consumption (with similar sensitivities) compared to MOS modalities, but electrochemical sensors suffer from limited operational lifespans and are highly sensitive to environmental fluctuations such as humidity and temperature.^6^

Optical sensors, such as surface plasmon resonance (SPR)^7^ and nondispersive infrared (NDIR) sensors,^8^ have the key advantage of using light that interacts directly with the target gas to conduct their measurements. Optics offers a number of advantages over the previous sensor modalities, such as superior selectivity and specificity, enhanced stability, longer operational lifespans, and reduced susceptibility to chemical poisoning and electromagnetic interference.

NDIR sensors measure the change in absorption of an infrared beam as it passes through a sample tube, providing high performance in both sensitivity and selectivity. However, NDIR intrinsically relies on long interaction lengths, making the devices large and cumbersome and unavailable for miniaturized or handheld/integrated formats.

Surface plasmon resonance sensors measure small changes in refractive index near their surface upon the binding of analytes and have been successfully exploited to make both biosensors and gas sensors.^9,10^ SPR sensors feature high sensitivity and can be easily functionalized to aid specificity; however, the intrinsic optical losses of plasmons suggest that their performance is inferior to all-dielectric resonant structures,^11^ which we are aiming to explore here.

Accordingly, we demonstrate that a chirped guided-mode resonance-based sensor, functionalized with a calixarene layer, can exceed the requirements for monitoring acetone in an industrial environment, while also overcoming key limitations of current technologies, such as high power consumption, high-temperature operation, and long-term drift.

### Functionalized Guided-Mode Resonance Sensors

#### Guided-Mode Resonance Sensors

Guided-mode resonance (GMR) sensors consist of a wavelength-scale diffraction grating that supports a waveguide mode. When illuminated, the grating diffracts light into the thin film at a resonant wavelength and polarization that corresponds to the first-order diffraction that phase-matches a resonant mode propagating along the waveguiding grating. Light scattered in-plane forms a standing wave, and light scattered out-of-plane in the forward direction interferes with the incoming light, creating destructive interference, leading to a 100% reflectance (in principle) from the grating. Similar to surface plasmon resonances (SPRs),^10^ the wavelength at which these conditions are met is highly sensitive to the refractive index of the cladding material. Since GMRs are realized in intrinsically lossless dielectric materials, however, their figure of merit for sensing (Q-factor × Sensitivity × Amplitude)^11^ tends to be higher than that of SPRs.

Typically, a spectrometer is required to monitor a photonic resonance; however, by using a chirped approach, whereby the grating period increases along the length of the sensor chip, spectral information can be converted into a spatial position.^12^ This is revealed as a bright resonance line along a grating, which, for a fixed wavelength, will shift up or down with a change in refractive index (Figure 1). Using this method, the tiny changes produced by target analyte binding can be measured using a simple, inexpensive camera.

**Figure 1:**
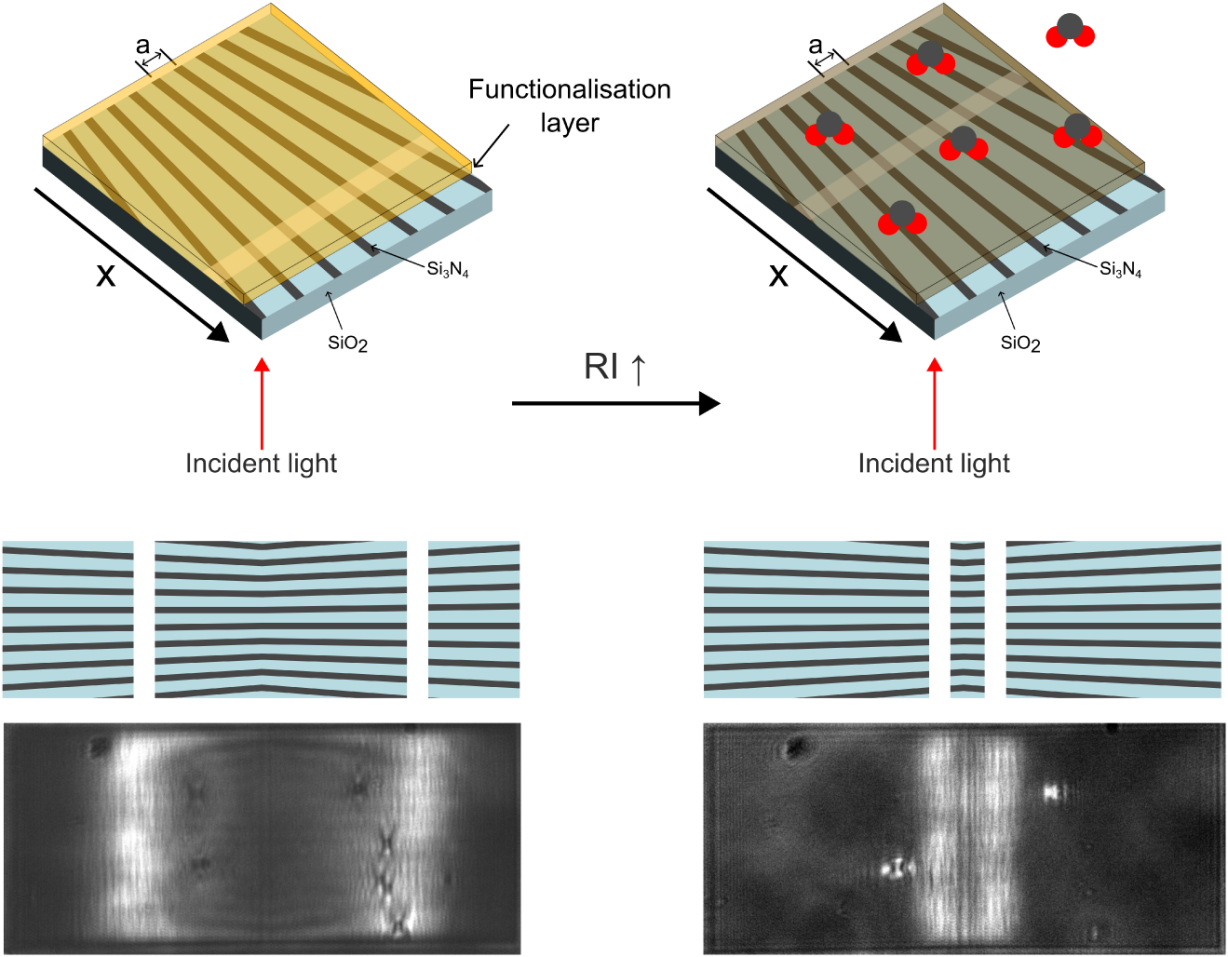
Schematic illustration of the operation of a functionalized chirped guided-mode resonance sensor. A change in the refractive index of the functionalization layer caused by the interaction with target molecules leads to a shift in the position of the resonance line. Experimental micrographs are shown in the bottom panel.

The sensors themselves are easy to mass-produce using existing lithography methods such as deep ultraviolet (DUV) lithography, making them readily scalable and offering lower costs at scale. Finally, GMRs can be designed to be insensitive to temperature and vibration interference, as we have recently shown in ref. 13.

#### Calixarenes

Calixarenes are macrocyclic compounds formed by the condensation of phenols with formaldehyde.^14^ They have attracted attention in recent years for their potential in sensing applications due to their well-established host–guest chemistry. Their three-dimensional basket-like shape features a central cavity, which can reversibly capture guest molecules, such as VOCs, through noncovalent interactions. Calixarenes are highly versatile, as their cavity size can be adjusted to tailor their selectivity toward differently sized molecules^15^ and functional groups, such as hydroxyls, can easily be added to enhance binding and solubility. Furthermore, calixarenes typically exhibit strong thermal stability, ensuring robustness in industrial settings.^16^

Heterocalixarenes are a subclass of calixarenes where the aromatic rings are bridged by a heteroatom.^17^ Here, we specifically consider an oxygen-bridged calixarene known as an oxacalixarene.^18^ Previous studies have shown that this class of molecules has a strong affinity for binding with VOCs; specifically, 2,14-dihydroxy-tetranitrooxacalix[4]­arene, herein known as DHTNOC, has been shown to be particularly selective toward acetone over other VOCs.^19^ Therefore, using DHTNOC provides a specificity for acetone detection. Figure 2 shows the chemical structure of DHTNOC as well as experimental crystallographic results that demonstrate its capability for specific hydrogen bonding with acetone.

**Figure 2:**
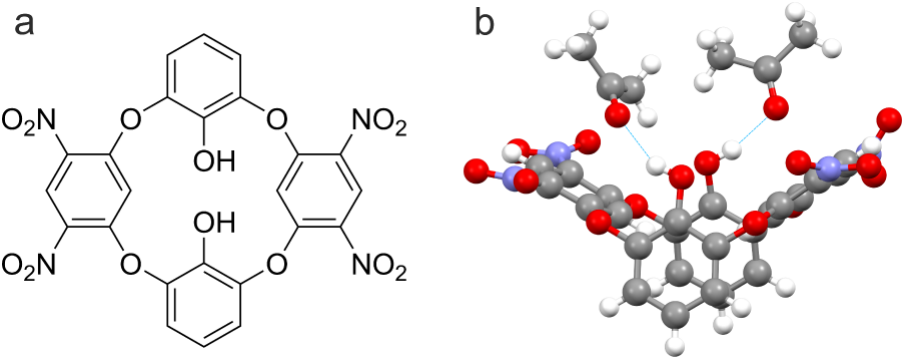
(a) Chemical structure of 2,14-dihydroxy-tetranitrooxacalix[4]­arene (DHTNOC). (b) Reconstructed crystallography data showing acetone forming hydrogen bonds with DHTNOC.

Calixarene thin films form a supramolecular lattice containing both intra- and extramolecular voids, where the former consists of the cup of an individual molecule and the latter, the spaces in between. These cavities act as a sponge toward VOCs, exhibiting pore filling upon interaction.^20^ As the pores are filled, air is replaced with higher-index VOCs, leading to an increase in the refractive index, which is what we observe. Film swelling also occurs, acting to reduce the refractive index; however, this effect is outweighed by increases due to pore filling. At higher concentrations, capillary condensation effects also take place, raising the index dramatically due to increased film density, which is particularly relevant for acetone due to its high volatility.^21,22^

## Experimental Details

### Calixarene Synthesis

DHTNOC was synthesized using a method similar to that described in Katz et al. (2005).^23^ Under ambient conditions, 1,5-difluoro-2,4-dinitrobenzene (400 mg, 1.96 mmol), 1,2,3-trihydroxybenzene (247 mg, 1.96 mmol), and finely ground potassium carbonate (677 mg, 4.90 mmol) are added to a 100 mL round-bottom flask. Anhydrous dimethyl sulfoxide (DMSO) (20 mL) is then added, and the resulting suspension is stirred vigorously at room temperature for 20 h. The reaction mixture is transferred to a separating funnel, where ethyl acetate (150 mL) is added, and the mixture is washed sequentially with 1 M aqueous HCl (50 mL) and brine (50 mL). The organic extracts are collected, dried over sodium sulfate, filtered, and concentrated in vacuo. The crude residue is then dissolved in a small amount of 1:1 DCM:MeOH, silica (∼2 g), and the residue is concentrated in vacuo to form a fine powder, which is used to dry-load the product during column chromatography. The silica/product mixed powder is dry-loaded onto a silica column and eluted with 100:1 to 50:1 DCM:MeOH. Collection of the pure fractions (analyzed by TLC) affords DHTNOC as a pale-yellow solid (273 mg, 24%).

### Surface Functionalization

To functionalize the surface of the GMR sensors, we first cleaned the samples with acetone and isopropyl alcohol (IPA) in an ultrasonic bath, followed by oxygen plasma treatment. We note that performing silanization of the surface with (3-aminopropyl)­triethoxysilane (APTES) helps with film formation. The silanization is achieved by submerging the sensors overnight in a 4% by volume solution of APTES in IPA before being rinsed with IPA. DHTNOC is dissolved in acetone before a thin film is deposited onto the sensor surface via spin coating at 4500 rpm. We experimented with other solvents for dissolving DHTNOC, such as anisole and ethanol, but none were as effective as acetone; in fact, this shows the high solubility of DHTNOC in acetone. Immediately after coating, we bake the sensor at 65 °C for 7 min to remove any remaining acetone. DHTNOC produces a microcrystalline film with an average thickness of (110 ± 5) nm, as measured using a Filmetrics F20 thin-film reflectometer. The quality of the films is verified visually under a microscope. The films that produced the greatest response to acetone were those that featured the highest level of uniformity, suggesting small crystal sizes. For films that did not produce good-quality resonances, we saw larger, micrometer-scale crystals on the surface. We suggest that these larger crystals induce greater scattering of light within the film, which leads to diminished resonances and a lower signal-to-noise ratio. Larger crystals also sit further outside the evanescent tail of the guided mode. We found that it was possible to reduce the crystal size by ensuring surface cleanliness and heating the DHTNOC solution in an ultrasonic bath before spinning. Any amount of dirt on the surface of the chip would provide the calixarene a location to bind to and agglomerate, resulting in larger crystals upon drying. Similarly, heating the solution in an ultrasonic bath aided in breaking apart undissolved DHTNOC within the solution, further reducing sites where crystals could grow larger upon spinning. Correspondingly, increasing the DHTNOC concentration also made film formation more difficult due to increased crystal agglomeration as the solution became saturated, and therefore, the concentration was capped at 13 mg/mL.

### Vapor-Sensing Experiments

We conducted vapor-sensing experiments in a bespoke gas cell designed to seal around the GMR sensing chip. The sensors were placed at the bottom of the cell and were illuminated from below by 647 nm light at normal incidence. We recorded the resonance line shift by projecting an image of the sensor chip onto a CMOS camera with an approximately 1:1 magnification. Figure 3 shows a schematic of the experimental setup.

**Figure 3:**
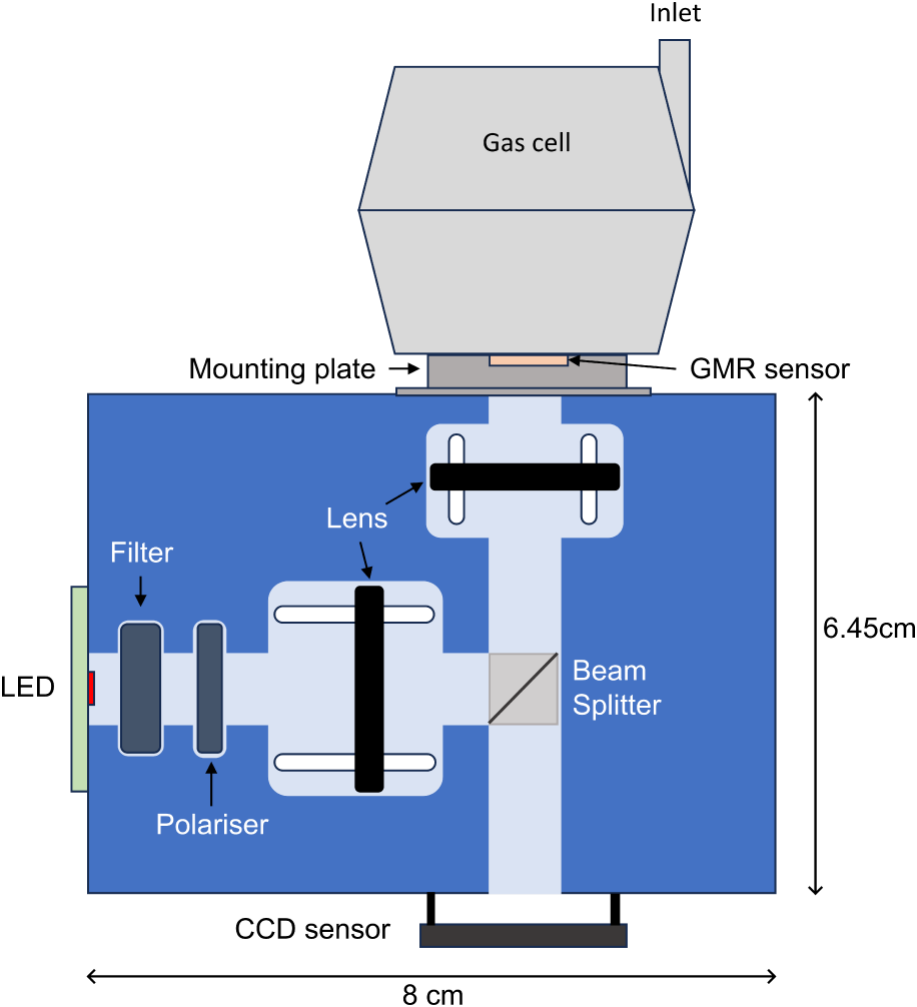
A schematic of the experimental setup, including both the optical components and the mounted gas cell (not to scale).

Creating a controlled concentration of acetone gas is not trivial, especially if the concentration is low. To this end, we injected microliter volumes of liquid acetone into a chamber of known volume. Injection occurs through a self-sealing membrane to reduce any losses out of the chamber. As the acetone enters the chamber, it forms a spray and evaporates almost instantaneously. We then used eq 1 to determine the concentration, whereby *C* (ppm) is the vapor concentration in parts per million, ρ the vapor density (0.79 g/mL for acetone), *V* the volume injected in microliters, *R* the universal gas constant (8.3145 J/(mol·K)), *T* the room temperature in Kelvin, *M* the molecular weight (58.08 g/mol for acetone), *P* the pressure in Pascals, and *V*_*c*_ the volume of the chamber in liters.

1 $$C\left(ppm\right)=\frac{\rho VRT}{MPV_{c}}\times 10^{6}$$

The sensor was exposed to the vapor for 70 s before the chamber was removed. In between each experiment, the chamber, without the sensor inside, was evacuated with nitrogen and placed in an oven at 65 °C for 5–10 min for outgassing. For cross-sensitivity experiments, this same method was used for ethanol and methanol.

## Results

The GMR sensor chips we used were produced in a silicon photonics foundry using 193 nm DUV lithography on a quartz substrate with a 150 nm thick silicon nitride waveguide layer. The sensors were designed by our research group and feature an 8 nm double-chirped “bowtie” design (Figure 1). Each half of the bowtie consisted of a 500 × 300 μm (L × W) grating, with a period increasing from 418 to 426 nm, alongside a constant fill factor of 63%, across the full 500 μm length. These gratings produce a GMR resonance with an evanescent tail extending approximately 180 nm into the superstrate.^13^ Before functionalizing, a bare chip response was recorded, which showed a sharp resonance at 647 nm in air. No resonance shift was observed upon exposure to acetone on the bare chip.

We tested the sensing performance by exposing the device to varying concentrations of acetone vapor. Figure 4a shows an example of a resonance shift upon exposure to 8300 ppm of acetone. The sensor responds very quickly, reaching a peak resonance shift in under 4 s, which we took as the performance metric. The response then decreases monotonically; upon removal of the chamber and exposure to air, the resonance returns to its initial position. We chose 8300 ppm as a convenient measure, as it corresponds to 10 μL of acetone evaporated into the 395 cm^3^ volume of our sample chamber.

**Figure 4:**
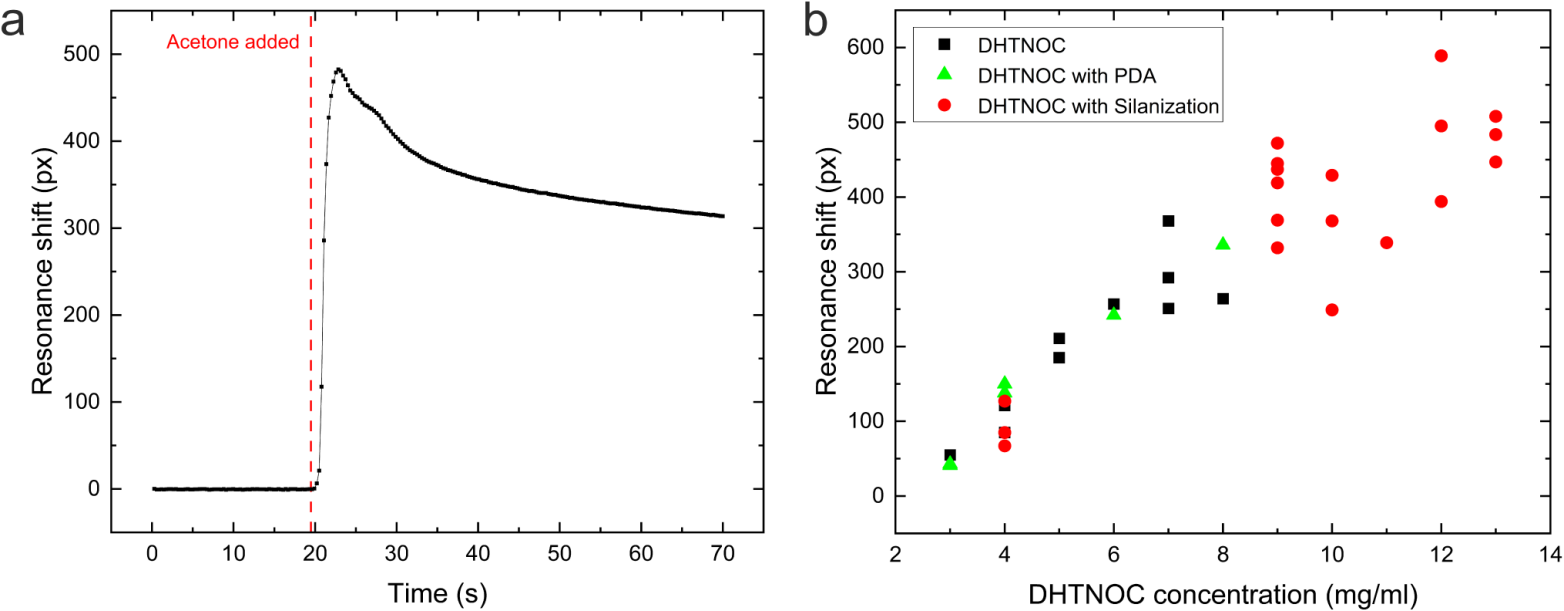
(a) Response curve of a DHTNOC-functionalized GMR sensor for 8300 ppm acetone. (b) Resonance shifts achieved for 8300 ppm exposure to acetone for films made with different DHTNOC concentrations and surface treatments.

Since the DHTNOC properties clearly determine the sensor’s response to acetone, we investigated the effect of the concentration of the DHTNOC solution used to produce the thin films, shown in Figure 4b. Each data point represents an exposure to 8300 ppm, and we observe the clear trend that the response scales with concentration. However, with an increase in the DHTNOC concentration, the films became much harder to produce uniformly, and their overall quality decreased. This reduction in the film quality is apparent from the increased variance between the data points. Beyond a concentration of 8 mg/mL, we noted that DHTNOC alone (black square points) no longer produces clear resonances, which we attribute to increased scattering due to the formation of larger microcrystals. To address this issue, we hypothesized that providing a surface attachment opportunity would offer the DHTNOC molecules an opportunity for forming a more uniform film and preventing them from crystallizing. We used both polydopamine (PDA) (green triangles) and APTES (red circles) because they both form thin films with high binding affinity. We found that PDA makes little difference to the film formation, while salinization with APTES helped improve uniformity and allowed us to achieve good resonances at higher concentrations. Beyond 13 mg/mL, however, the films became much harder to produce and yielded no further improvements; hence, we chose this value for the film concentration.

To determine the limit of detection of the system, the sensor was exposed to acetone concentrations between 780 and 6300 ppm; 780 ppm was the lowest concentration of acetone we could reliably achieve, so the LOD was determined by extrapolation against the noise level. Between measurements, the sensor was exposed to air to reset its resonance position. Figure 5 shows the response curve of the sensor as a function of the concentration.

**Figure 5:**
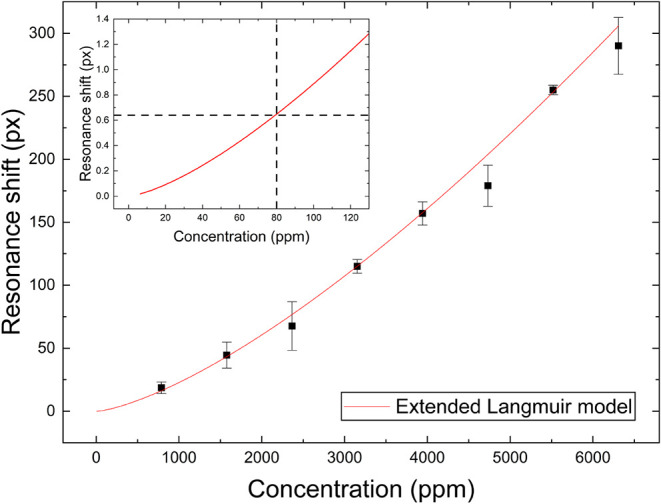
Response curve of the sensor as a function of the acetone concentration. The curve fits well to the extended Langmuir model, with a limit of detection estimated to be 80 ppm (inset).

The sensor exhibits a nonlinear response curve that is well described by an extended Langmuir model with an R-squared value greater than 0.99. The model is summarized by eq 2, whereby *q* is the amount of adsorption per unit weight of the adsorbate, *Q*_*m*_ the adsorption capacity of the system, *c* the concentration of the adsorbent, *k* the affinity constant, and *n* a dimensionless constant.

2 $$q=\frac{Q_{m}kc^{1-n}}{1+kc^{1-n}}$$

The model is based on the well-known Langmuir isotherm and then extends the shape-governing parameter from *k* to *kc* ^–*n*^, which varies with the adsorbent concentration.^24^ This model better describes adsorption onto nonuniform surfaces and takes into account surface occupancy.

The limit of detection of the sensor was taken as the point at which the fitted curve met the average 3σ noise value, which here is 0.64 pixels. This approach resulted in a limit of detection of 80 ppm.

An ideal sensor should be stable over extended periods of time and exhibit the same response to repeated exposures at the same concentrations. To test this capability, we exposed the sensor repeatedly (10×) to a concentration of 8300 ppm of acetone over a period of 50 min. Between each measurement, the sensor was exposed to air to reset its resonance position. Figure 6a shows the responses obtained. We observed a standard deviation of 17.0 pixels. Considering the average shift value of 380 pixels, this deviation is equivalent to a 4.5% error bar.

**Figure 6:**
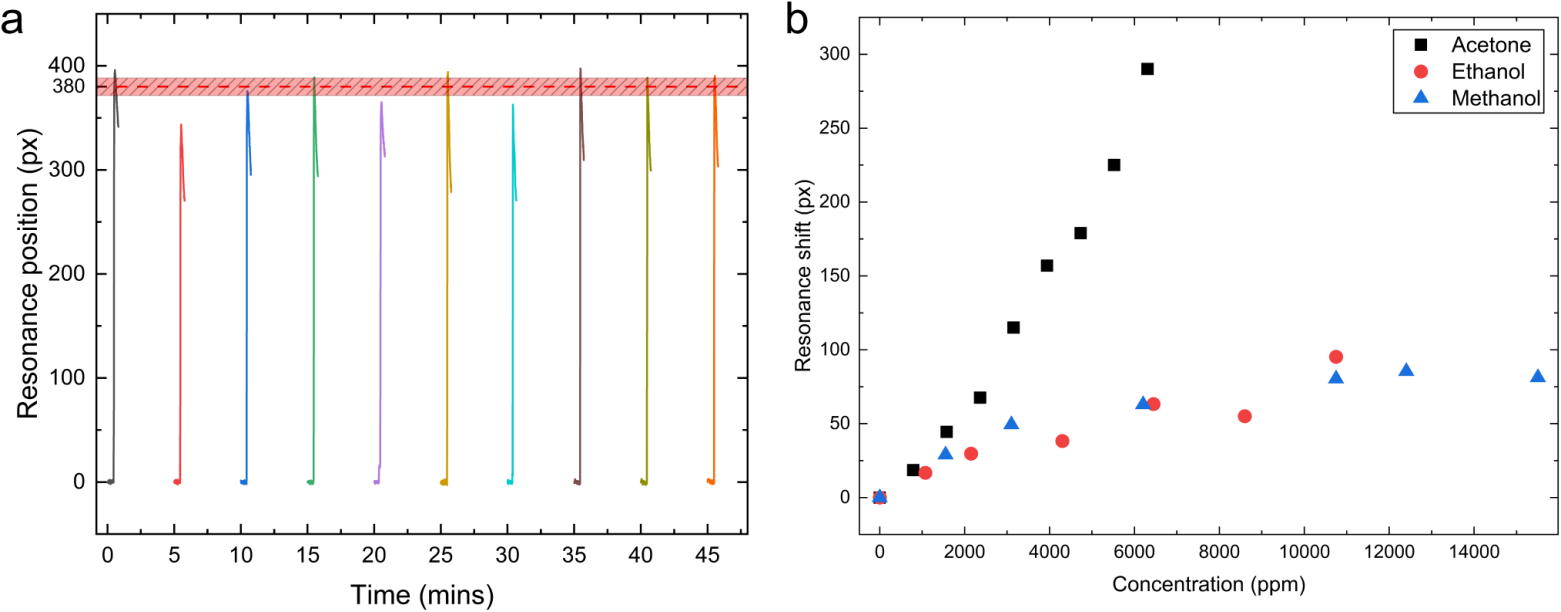
(a) Repeated exposures of the same sensor to 8300 ppm of acetone over 50 min. The average value of 380 pixels and standard deviation of 17.0 pixels (4.5%) are illustrated by the shaded box. (b) DHTNOC selectivity responses to competing VOCs.

To investigate the selectivity of the DHTNOC-coated sensor toward acetone against other common VOCs, the sensor was exposed to both ethanol and methanol over a range of concentrations (Figure 6b). While it is not possible to be completely selective, as all VOCs will interact with the sensor surface in some way, the results in Figure 6b show that DHTNOC features a significantly greater response to acetone over ethanol and methanol, two commonly competing VOCs.

## Discussion

The limit of detection of 80 ppm that we report is well below a typical workplace exposure limit. For example, the UK’s 8-hour work exposure limit is 500 ppm,^25^ and prolonged exposure to 173 ppm is known to cause harm.^1^ Being able to see <100 ppm with a simple instrument is therefore essential for the continuous monitoring and early detection of leaks and hazards in the workplace. While other modalities may exhibit lower limits of detection, we suggest that ours is a better fit for purpose. For example, MOS sensors achieve lower levels of detection (in the single-digit ppm range), but they require high operating temperatures with correspondingly high power consumption. In contrast, our method only uses low-power components, such as an LED light source and a CMOS camera, as shown in Figure 3, and requires no heating for operation. Moreover, as we have shown in ref. 13, it is suitable for operation in industrial environments and can compensate for temperature fluctuations.^13^

The LOD we achieve is attributed to both the sensitivity of the GMR platform and the calixarene functionalization layer. The all-dielectric GMR sensor provides a medium-quality resonance capable of measuring small spectral shifts, while the host–guest chemistry of the calixarene functionalization layer offers a preferential affinity for acetone molecules. The four rings of DHTNOC provide an ideal cavity size to capture one or two acetone molecules per calixarene, while the hydroxyl groups successfully form hydrogen bonds with the acetone. Together, these effects efficiently capture acetone on the sensor surface, increasing the local concentration and amplifying the refractive index change. While we note that DHTNOC has already previously been shown to be selective toward acetone.^19^

As acetone interacts with the sensor, it will have a dynamic equilibrium between adsorption and desorption on the calixarene layer. It is possible that this may lead to very small fluctuations in the refractive index. This, however, does not affect the signal, as the recorded system noise level remains constant both before and after acetone exposure. Any refractive index fluctuation as a result of the dynamic equilibrium is therefore below the noise level and not registered.

The low 4.5% standard deviation shows stability between individual measurements, which is important for reliability. The solid-state nature of the underlying GMR sensor is also immune to long-term drift. We note that the calixarene layer has been stable over a number of months in the laboratory, which suggests considerable long-term stability of this most sensitive aspect of the sensor. To support this observation, we note that calixarenes are known to exhibit good thermal stability.^16^

Our results establish the GMR-based sensor as a versatile platform for gas sensing beyond acetone and other single-species detection, for example, toward an “optical nose” application. The small size of the sensors and the possibility of functionalizing them in the style of a microarray make the GMR platform well suited for constructing sensor arrays. By spotting a series of cross-reactive bioreceptors onto individual gratings, for example, these arrays can then generate high-dimensional response “fingerprints”. Using suitable data analysis, these “fingerprints” can then be used to identify individual gases within complex mixtures or to recognize patterns or odors within. This capability opens up applications in industries such as scent control in food and perfume production as well as noninvasive medical diagnostics, an area that we are currently exploring via the use of peptides as cross-reactive arrays.^26^

## Conclusions

In summary, we have successfully demonstrated an optical acetone gas sensor created by combining a chirped guided-mode resonance (GMR) sensor with a selective calixarene functionalization layer. The sensor achieves a limit of detection of 80 ppm, which is highly relevant for monitoring in industrial applications, being far below, for example, the UK workplace safety limits. The sensor response curve fits well to an extended Langmuir isotherm, allowing quantification of acetone concentrations across a broad range of concentrations; and the sensors also feature a low standard deviation across measurements of 4.5%. The platform is competitive compared to other similarly performing modalities due to its room temperature, low power consumption, and operation, which does not require expensive spectrometers or cameras. This approach is highly versatile due to its simplicity and ease of functionalization thanks to its simple surface chemistry and has the possibility to be extended to sensing arrays for the measurement of complex gas mixtures. Ultimately, this work showcases the functionalized chirped-GMR platform as a powerful method for future next-generation gas sensors.

## Abbreviations

VOC: Volatile Organic Compound
GMR: Guided-Mode Resonance
DHTNOC: 2,14-dihydroxy-tetranitrooxacalix­[4]­arene
MOS: Metal-Oxide Sensor
SPR: Surface Plasmon Resonance
NDIR: Nondispersive Infrared
